# 1-[2-(Benzyl­amino)-4-pyrid­yl]-2-(4-fluoro­phen­yl)ethane-1,2-dione

**DOI:** 10.1107/S1600536809019801

**Published:** 2009-05-29

**Authors:** Hartmut Jahns, Pierre Koch, Dieter Schollmeyer, Stefan Laufer

**Affiliations:** a Institute of Pharmacy, Department of Pharmaceutical and Medicinal Chemistry, Eberhard-Karls-University Tübingen, Auf der Morgenstelle 8, 72076 Tübingen, Germany; b Department of Organic Chemistry, Johannes Gutenberg-University Mainz, Duesbergweg 10-14, D-55099 Mainz, Germany

## Abstract

The crystal structure of the title compound, C_20_H_15_FN_2_O_2_, contains two crystallographically independent mol­ecules, which are related by a pseudo-inversion center and linked into dimers *via* inter­molecular N—H⋯N hydrogen bonds. The 4-fluoro­phenyl ring of mol­ecule *A* makes dihedral angles of 17.17 (16) and 62.25 (15)°, respectively, with the phenyl and pyridine rings. The 4-fluoro­phenyl ring of mol­ecule *B* makes dihedral angles of 8.50 (16) and 64.59 (15)°, respectively, with the phenyl and pyridine rings. The dihedral angle between the pyridine ring and the phenyl ring of mol­ecule *A* [60.97 (15)°] is bigger than in mol­ecule *B* [59.49 (15)°]. The dihedral angle between the two pyridine rings is 1.37 (14)° and between the two phenyl rings is 3.64 (16)°.

## Related literature

For α-diketones as inter­mediates in the synthesis of heterocycles, see: Ohta *et al.* (1982 ▶); Wolkenberg *et al.* (2004 ▶); Zhao *et al.* (2003 ▶,2004 ▶).
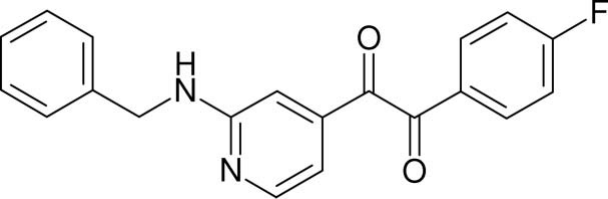


## Experimental

### 

#### Crystal data

C_20_H_15_FN_2_O_2_
*M* *_r_* = 334.34
Orthorhombic, 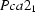

*a* = 12.0951 (13) Å
*b* = 7.4097 (8) Å
*c* = 35.163 (5) Å
*V* = 3151.3 (7) Å^3^
*Z* = 8
Cu *K*α radiation
μ = 0.83 mm^−1^
*T* = 193 K
0.38 × 0.19 × 0.10 mm

#### Data collection

Enraf–Nonius CAD-4 diffractometer
Absorption correction: none
5637 measured reflections
5558 independent reflections
4832 reflections with *I* > 2σ(*I*)
*R*_int_ = 0.054
3 standard reflections frequency: 60 min intensity decay: 2%

#### Refinement

*R*[*F* ^2^ > 2σ(*F* ^2^)] = 0.048
*wR*(*F* ^2^) = 0.132
*S* = 1.05
5558 reflections
451 parameters
1 restraint
H-atom parameters constrained
Δρ_max_ = 0.19 e Å^−3^
Δρ_min_ = −0.23 e Å^−3^
Absolute structure: Flack (1983 ▶), 2522 Friedel pairs
Flack parameter: 0.35 (19)

### 

Data collection: *CAD-4 Software* (Enraf–Nonius, 1989 ▶); cell refinement: *CAD-4 Software*; data reduction: *CORINC* (Dräger & Gattow, 1971 ▶); program(s) used to solve structure: *SIR97* (Altomare *et al.*, 1999 ▶); program(s) used to refine structure: *SHELXL97* (Sheldrick, 2008 ▶); molecular graphics: *PLATON* (Spek, 2009 ▶); software used to prepare material for publication: *PLATON* .

## Supplementary Material

Crystal structure: contains datablocks I, global. DOI: 10.1107/S1600536809019801/nc2145sup1.cif

Structure factors: contains datablocks I. DOI: 10.1107/S1600536809019801/nc2145Isup2.hkl

Additional supplementary materials:  crystallographic information; 3D view; checkCIF report

## Figures and Tables

**Table 1:** Hydrogen-bond geometry (Å, °)

*D*—H⋯*A*	*D*—H	H⋯*A*	*D*⋯*A*	*D*—H⋯*A*
N17—H17⋯N44	0.94	2.10	3.037 (3)	174
N47—H47⋯N14	0.94	2.15	3.087 (4)	172

**Fig. 1.**
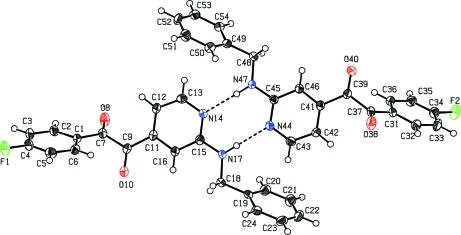
View of title compound with labeling. Displacement ellipsoids are drawn at the 50% probability level. H atoms are depicted as circles of arbitrary size. Hydrogen bonds with dashed lines.

**Fig. 2.**
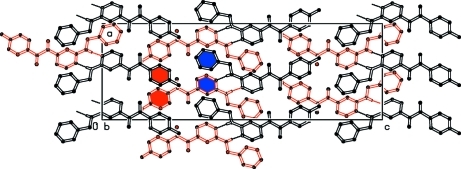
Crystal structure of the title compound. View along the b axis. Aromatic rings involved in π-π interactions are shown in red and blue.

## supplementary crystallographic information

### Comment

Substituted α-diketones are important building blocks for the synthesis of a variety of heterocyclic compounds, like 1,2,4-triazines (Zhao *et al.* 2003), chinoxalines (Zhao *et al.* 2004), pyrimidines (Ohta *et al.* 1982) or imidazoles (Wolkenberg *et al.* 2004).

The title compound, 1-(2-(benzylamino)pyridin-4-yl)-2-(4-fluorophenyl)ethane-1,2-dione, was synthesized as an intermediate in the synthesis of 2-(2-alkylaminopyridin-4-yl)-3-(4-fluorophenyl)chinoxalines as potent p38 mitogen-activated protein (MAP) kinase inhibitors.

The crystal structure contains two crystallographically independent molecules of slightly different conformation (Fig. 1). The molecules are related by a pseudo inversion center which is not part of the space group and formes dimers due to intermolecular N–H···N hydrogen bonds (N17—H17···N44 2.10Å and N47—H47···N14 2.15 Å).

The 4-fluorophenyl ring (C1—C6) of molecule A makes dihedral angles of 17.17 (16)° and 62.25 (15)° to the phenyl ring (C19—C24) and the pyridine ring (C11—C13,N14,C15—C16), respectively. The 4-fluorophenyl ring (C31—C36) of molecule B makes dihedral angles of 8.50 (16)° and 64.59 (15)° to the phenyl ring (C49—C54) and the pyridine ring (C41—C43,N44,C45—C46), respectively. The dihedral angle between the pyridine ring and the phenyl ring of molecule A [60.97 (15)°] is bigger than in molecule B [59.49 (15)°]. The dihedral angle between the two pyridine rings is 1.37 (14)° and between the two phenyl rings is 3.64 (16)°. The two molecules forms π-π interaction between the phenyl rings C1—C6/C31–36 (red in Fig.2) and phenyl/pyridin rings C19—C24/C41—C46 (blue in Fig.2). The distances between the centroids C1—C6···C31—C36 and C19—C24···C41—C46 are 3.81Å and 3.83 Å, respectively. The least square planes through the corresponding rings have angles of 2.04° and 9.8°.

### Experimental

Selenium dioxide (0.64 g, 5.7 mmol) and *tert*-butyl *N*-benzyl-*N*-[4-(4-fluorobenzoylmethyl)- 2-pyridyl]carbamate (2.0 g, 4.8 mmol) were treated with glacial acetic acid (40 ml) and heated 4.5 h to reflux temperature. After cooling to room temperature water (70 ml) was added and selenium was filtered off. Ethyl acetate was added to the filtrate and the aqueous phase was extracted twice with ethyl acetate. The combined organic phases were evaporated and the residue was purified by flash-chromatography (silica gel, petroleum ether - ethyl acetate 4:1 to 3:1) to yield 0.82 g (51%) as a red solid. Crystals of the title compound suitable for X-ray diffraction were obtained by slow evaporation of a solution of the solid in ethyl acetate at 298 K.

### Refinement

Reflections were measured in the range 0 – +h, 0 – +k, 0 – +l and Friedel pairs. Hydrogen atoms attached to carbons were placed at calculated positions with C—H = 0.95 Å (aromatic) or 0.98–0.99 Å (*sp*^3^ C-atom). All H atoms were refined in the riding-model approximation with isotropic displacement parameters (set at 1.2–1.5 times of the *U*_eq_ of the parent atom). Hydrogen atoms attached to N17 and N47 were located in diff. Fourier maps and refined with fixed isotropic displacement parameters and applying riding motion model. The absolute structure cannot be determined with certainty and refinement of the structure as a racemic twin does not lead to any improvement of the reliability factors.

### Crystal data

**Table:**

C_20_H_15_FN_2_O_2_	*F*(000) = 1392
*M**_r_* = 334.34	*D*_x_ = 1.409 Mg m^−^^3^
Orthorhombic, *P**c**a*2_1_	Cu *K*α radiation, λ = 1.54178 Å
Hall symbol: P 2c -2ac	Cell parameters from 25 reflections
*a* = 12.0951 (13) Å	θ = 30–46°
*b* = 7.4097 (8) Å	µ = 0.83 mm^−^^1^
*c* = 35.163 (5) Å	*T* = 193 K
*V* = 3151.3 (7) Å^3^	Plate, light brown
*Z* = 8	0.38 × 0.19 × 0.10 mm

### Data collection

**Table:**

Enraf–Nonius CAD-4 diffractometer	*R*_int_ = 0.054
Radiation source: FR571 rotating anode	θ_max_ = 70.0°, θ_min_ = 2.5°
graphite	*h* = −14→14
ω/2θ scans	*k* = −9→9
5637 measured reflections	*l* = −42→42
5558 independent reflections	3 standard reflections every 60 min
4832 reflections with *I* > 2σ(*I*)	intensity decay: 2%

### Refinement

**Table:**

Refinement on *F*^2^	Secondary atom site location: difference Fourier map
Least-squares matrix: full	Hydrogen site location: inferred from neighbouring sites
*R*[*F*^2^ > 2σ(*F*^2^)] = 0.048	H-atom parameters constrained
*wR*(*F*^2^) = 0.132	*w* = 1/[σ^2^(*F*_o_^2^) + (0.079*P*)^2^ + 0.0063*P*] where *P* = (*F*_o_^2^ + 2*F*_c_^2^)/3
*S* = 1.05	(Δ/σ)_max_ < 0.001
5558 reflections	Δρ_max_ = 0.19 e Å^−^^3^
451 parameters	Δρ_min_ = −0.23 e Å^−^^3^
1 restraint	Absolute structure: Flack (1983), 2522 Friedel pairs
Primary atom site location: structure-invariant direct methods	Flack parameter: 0.35 (19)

### Special details

**Table:**

Geometry. All e.s.d.'s (except the e.s.d. in the dihedral angle between two l.s. planes) are estimated using the full covariance matrix. The cell e.s.d.'s are taken into account individually in the estimation of e.s.d.'s in distances, angles and torsion angles; correlations between e.s.d.'s in cell parameters are only used when they are defined by crystal symmetry. An approximate (isotropic) treatment of cell e.s.d.'s is used for estimating e.s.d.'s involving l.s. planes.
Refinement. Refinement of *F*^2^ against ALL reflections. The weighted *R*-factor *wR* and goodness of fit *S* are based on *F*^2^, conventional *R*-factors *R* are based on *F*, with *F* set to zero for negative *F*^2^. The threshold expression of *F*^2^ > σ(*F*^2^) is used only for calculating *R*-factors(gt) *etc*. and is not relevant to the choice of reflections for refinement. *R*-factors based on *F*^2^ are statistically about twice as large as those based on *F*, and *R*- factors based on ALL data will be even larger.

### Fractional atomic coordinates and isotropic or equivalent isotropic displacement parameters (Å^2^)

**Table:**

	*x*	*y*	*z*	*U*_iso_*/*U*_eq_	
F1	0.35951 (18)	0.4513 (3)	0.22618 (6)	0.0511 (6)	
C1	0.5181 (2)	0.3936 (4)	0.32829 (8)	0.0219 (6)	
C2	0.5644 (3)	0.3233 (4)	0.29506 (8)	0.0261 (6)	
H2	0.6328	0.2605	0.2963	0.031*	
C3	0.5120 (3)	0.3438 (5)	0.26040 (9)	0.0321 (8)	
H3	0.5437	0.2974	0.2377	0.038*	
C4	0.4121 (3)	0.4340 (5)	0.25979 (8)	0.0315 (7)	
C5	0.3636 (3)	0.5030 (5)	0.29206 (9)	0.0308 (7)	
H5	0.2946	0.5640	0.2906	0.037*	
C6	0.4166 (2)	0.4825 (4)	0.32648 (8)	0.0253 (6)	
H6	0.3839	0.5289	0.3490	0.030*	
C7	0.5813 (2)	0.3725 (4)	0.36420 (8)	0.0233 (6)	
O8	0.67739 (19)	0.3251 (3)	0.36487 (6)	0.0356 (6)	
C9	0.5196 (3)	0.3942 (4)	0.40219 (8)	0.0240 (6)	
O10	0.42846 (19)	0.3260 (3)	0.40449 (6)	0.0356 (6)	
C11	0.5752 (3)	0.4838 (4)	0.43500 (8)	0.0221 (6)	
C12	0.6723 (2)	0.5836 (4)	0.43184 (8)	0.0229 (6)	
H12	0.7112	0.5924	0.4084	0.027*	
C13	0.7097 (2)	0.6697 (4)	0.46454 (8)	0.0241 (6)	
H13	0.7738	0.7430	0.4624	0.029*	
N14	0.6625 (2)	0.6572 (3)	0.49862 (7)	0.0230 (5)	
C15	0.5701 (2)	0.5550 (4)	0.50181 (8)	0.0223 (6)	
C16	0.5233 (3)	0.4685 (4)	0.47028 (8)	0.0239 (6)	
H16	0.4572	0.4003	0.4729	0.029*	
N17	0.5271 (2)	0.5395 (4)	0.53755 (6)	0.0249 (6)	
H17	0.5433	0.6356	0.5543	0.030*	
C18	0.4209 (3)	0.4514 (4)	0.54384 (8)	0.0253 (6)	
H18A	0.4219	0.3300	0.5321	0.030*	
H18B	0.3616	0.5226	0.5316	0.030*	
C19	0.3972 (2)	0.4342 (4)	0.58592 (8)	0.0235 (6)	
C20	0.4743 (3)	0.3576 (5)	0.61064 (9)	0.0301 (7)	
H20	0.5423	0.3131	0.6008	0.036*	
C21	0.4531 (3)	0.3457 (5)	0.64931 (9)	0.0335 (7)	
H21	0.5065	0.2950	0.6660	0.040*	
C22	0.3528 (3)	0.4088 (5)	0.66351 (8)	0.0318 (7)	
H22	0.3377	0.4022	0.6900	0.038*	
C23	0.2759 (3)	0.4803 (5)	0.63923 (9)	0.0325 (8)	
H23	0.2074	0.5225	0.6490	0.039*	
C24	0.2967 (3)	0.4919 (4)	0.60057 (9)	0.0261 (6)	
H24	0.2420	0.5396	0.5840	0.031*	
F2	0.88809 (18)	0.7836 (3)	0.85731 (5)	0.0467 (5)	
C31	0.7257 (3)	0.9818 (4)	0.76425 (7)	0.0233 (6)	
C32	0.6788 (3)	0.9983 (5)	0.80060 (8)	0.0287 (7)	
H32	0.6093	1.0562	0.8036	0.034*	
C33	0.7337 (3)	0.9305 (5)	0.83209 (8)	0.0332 (7)	
H33	0.7032	0.9422	0.8569	0.040*	
C34	0.8330 (3)	0.8461 (4)	0.82649 (9)	0.0313 (7)	
C35	0.8795 (3)	0.8194 (4)	0.79135 (9)	0.0290 (7)	
H35	0.9466	0.7542	0.7886	0.035*	
C36	0.8256 (2)	0.8907 (4)	0.75999 (8)	0.0241 (6)	
H36	0.8570	0.8772	0.7354	0.029*	
C37	0.6681 (2)	1.0626 (4)	0.73148 (8)	0.0241 (6)	
O38	0.57767 (19)	1.1330 (4)	0.73326 (6)	0.0361 (6)	
C39	0.7292 (2)	1.0728 (4)	0.69328 (8)	0.0222 (6)	
O40	0.81709 (18)	1.1522 (3)	0.69255 (6)	0.0307 (5)	
C41	0.6762 (3)	0.9946 (4)	0.65860 (8)	0.0213 (6)	
C42	0.5787 (2)	0.8949 (4)	0.66072 (8)	0.0238 (6)	
H42	0.5397	0.8820	0.6840	0.029*	
C43	0.5413 (3)	0.8159 (4)	0.62775 (9)	0.0246 (6)	
H43	0.4759	0.7452	0.6291	0.030*	
N44	0.5911 (2)	0.8321 (3)	0.59388 (6)	0.0228 (5)	
C45	0.6849 (2)	0.9320 (4)	0.59180 (8)	0.0208 (6)	
C46	0.7304 (2)	1.0139 (4)	0.62435 (8)	0.0220 (6)	
H46	0.7971	1.0812	0.6227	0.026*	
N47	0.7298 (2)	0.9502 (4)	0.55656 (7)	0.0251 (6)	
H47	0.7030	0.8676	0.5383	0.030*	
C48	0.8360 (3)	1.0397 (4)	0.55111 (8)	0.0261 (7)	
H48A	0.8950	0.9675	0.5634	0.031*	
H48B	0.8343	1.1597	0.5635	0.031*	
C49	0.8623 (3)	1.0622 (4)	0.50941 (8)	0.0239 (6)	
C50	0.7872 (3)	1.1396 (4)	0.48458 (9)	0.0287 (7)	
H50	0.7173	1.1786	0.4938	0.034*	
C51	0.8132 (3)	1.1606 (5)	0.44644 (9)	0.0328 (8)	
H51	0.7608	1.2127	0.4296	0.039*	
C52	0.9152 (3)	1.1059 (5)	0.43283 (8)	0.0328 (7)	
H52	0.9327	1.1203	0.4067	0.039*	
C53	0.9910 (3)	1.0306 (5)	0.45723 (9)	0.0321 (7)	
H53	1.0611	0.9932	0.4480	0.038*	
C54	0.9651 (3)	1.0090 (4)	0.49550 (9)	0.0261 (6)	
H54	1.0179	0.9576	0.5123	0.031*	

### Atomic displacement parameters (Å^2^)

**Table:**

	*U*^11^	*U*^22^	*U*^33^	*U*^12^	*U*^13^	*U*^23^
F1	0.0571 (13)	0.0679 (15)	0.0282 (10)	−0.0039 (12)	−0.0212 (9)	0.0025 (10)
C1	0.0243 (14)	0.0239 (14)	0.0174 (13)	−0.0046 (12)	−0.0001 (11)	−0.0020 (11)
C2	0.0266 (15)	0.0285 (17)	0.0231 (14)	−0.0008 (13)	−0.0003 (12)	−0.0061 (12)
C3	0.0363 (17)	0.040 (2)	0.0198 (14)	−0.0057 (16)	0.0019 (12)	−0.0097 (13)
C4	0.0331 (17)	0.0404 (19)	0.0209 (14)	−0.0056 (16)	−0.0086 (12)	0.0032 (13)
C5	0.0251 (15)	0.0329 (16)	0.0344 (16)	0.0012 (15)	−0.0044 (13)	0.0034 (14)
C6	0.0269 (15)	0.0262 (15)	0.0228 (13)	0.0013 (13)	0.0029 (12)	−0.0015 (12)
C7	0.0258 (15)	0.0239 (14)	0.0202 (13)	0.0004 (13)	−0.0016 (12)	−0.0010 (11)
O8	0.0295 (12)	0.0499 (15)	0.0274 (11)	0.0116 (11)	−0.0028 (9)	−0.0086 (10)
C9	0.0271 (16)	0.0270 (16)	0.0180 (13)	−0.0007 (13)	0.0000 (11)	0.0017 (12)
O10	0.0352 (12)	0.0522 (15)	0.0196 (10)	−0.0137 (12)	−0.0018 (9)	−0.0015 (10)
C11	0.0261 (15)	0.0224 (14)	0.0179 (13)	0.0045 (13)	−0.0004 (12)	−0.0005 (11)
C12	0.0247 (14)	0.0268 (15)	0.0171 (12)	0.0031 (13)	−0.0002 (11)	0.0013 (11)
C13	0.0200 (14)	0.0240 (15)	0.0283 (15)	0.0005 (13)	0.0003 (11)	0.0024 (11)
N14	0.0232 (12)	0.0268 (13)	0.0189 (11)	−0.0033 (11)	−0.0013 (10)	−0.0017 (10)
C15	0.0226 (14)	0.0247 (15)	0.0196 (13)	0.0042 (13)	−0.0017 (11)	0.0011 (11)
C16	0.0273 (16)	0.0257 (15)	0.0188 (14)	−0.0023 (14)	−0.0014 (12)	0.0009 (11)
N17	0.0278 (14)	0.0298 (14)	0.0170 (11)	−0.0101 (12)	0.0006 (10)	−0.0039 (10)
C18	0.0254 (15)	0.0288 (15)	0.0218 (14)	−0.0084 (14)	−0.0013 (12)	−0.0034 (12)
C19	0.0268 (15)	0.0214 (14)	0.0222 (14)	−0.0076 (13)	−0.0016 (11)	−0.0018 (11)
C20	0.0310 (16)	0.0294 (17)	0.0298 (16)	0.0001 (15)	0.0028 (13)	−0.0002 (13)
C21	0.0391 (19)	0.0320 (18)	0.0295 (17)	0.0012 (16)	−0.0033 (14)	0.0057 (13)
C22	0.0425 (19)	0.0313 (18)	0.0216 (15)	−0.0010 (16)	0.0073 (13)	0.0006 (12)
C23	0.0307 (18)	0.0341 (18)	0.0327 (16)	−0.0034 (15)	0.0110 (14)	−0.0031 (14)
C24	0.0232 (15)	0.0265 (15)	0.0286 (15)	−0.0029 (14)	−0.0010 (13)	0.0008 (12)
F2	0.0580 (13)	0.0545 (13)	0.0275 (10)	0.0088 (11)	−0.0058 (9)	0.0144 (9)
C31	0.0265 (15)	0.0262 (15)	0.0173 (13)	−0.0051 (13)	0.0002 (11)	−0.0043 (11)
C32	0.0285 (16)	0.0333 (17)	0.0244 (15)	0.0011 (14)	0.0055 (12)	−0.0046 (13)
C33	0.046 (2)	0.0357 (17)	0.0182 (14)	−0.0005 (16)	0.0047 (13)	0.0008 (13)
C34	0.0433 (18)	0.0268 (16)	0.0238 (14)	−0.0042 (15)	−0.0058 (14)	0.0071 (12)
C35	0.0258 (16)	0.0318 (17)	0.0293 (15)	0.0012 (14)	0.0018 (12)	0.0024 (13)
C36	0.0264 (15)	0.0261 (15)	0.0199 (13)	−0.0029 (13)	0.0025 (11)	−0.0016 (11)
C37	0.0275 (15)	0.0261 (15)	0.0186 (13)	−0.0010 (14)	0.0008 (11)	−0.0060 (11)
O38	0.0307 (12)	0.0518 (15)	0.0257 (11)	0.0097 (12)	−0.0008 (9)	−0.0047 (10)
C39	0.0233 (14)	0.0258 (15)	0.0175 (13)	0.0015 (13)	0.0008 (11)	0.0016 (11)
O40	0.0295 (11)	0.0392 (13)	0.0234 (10)	−0.0109 (10)	−0.0032 (8)	0.0005 (9)
C41	0.0221 (14)	0.0238 (14)	0.0180 (13)	0.0022 (12)	−0.0018 (11)	0.0010 (11)
C42	0.0235 (14)	0.0269 (15)	0.0210 (13)	−0.0013 (13)	0.0036 (11)	−0.0012 (11)
C43	0.0218 (14)	0.0259 (15)	0.0263 (14)	−0.0019 (13)	−0.0017 (11)	0.0000 (12)
N44	0.0232 (12)	0.0258 (13)	0.0192 (11)	−0.0002 (11)	−0.0003 (9)	−0.0030 (9)
C45	0.0238 (14)	0.0195 (13)	0.0190 (13)	−0.0019 (13)	−0.0027 (11)	0.0012 (11)
C46	0.0214 (15)	0.0252 (15)	0.0195 (13)	−0.0010 (12)	0.0002 (12)	−0.0012 (11)
N47	0.0298 (14)	0.0300 (13)	0.0155 (10)	−0.0111 (12)	0.0012 (10)	−0.0016 (10)
C48	0.0286 (16)	0.0283 (16)	0.0213 (15)	−0.0082 (14)	0.0004 (12)	−0.0038 (12)
C49	0.0282 (16)	0.0200 (14)	0.0235 (14)	−0.0055 (14)	0.0024 (11)	−0.0023 (11)
C50	0.0284 (16)	0.0294 (16)	0.0284 (15)	0.0006 (14)	0.0014 (12)	0.0018 (12)
C51	0.0393 (19)	0.0317 (18)	0.0275 (15)	−0.0003 (16)	−0.0068 (14)	0.0050 (13)
C52	0.0486 (19)	0.0331 (17)	0.0167 (13)	−0.0072 (17)	0.0071 (13)	−0.0014 (13)
C53	0.0294 (16)	0.0363 (18)	0.0304 (15)	−0.0082 (15)	0.0061 (13)	−0.0051 (14)
C54	0.0249 (15)	0.0273 (15)	0.0259 (14)	−0.0041 (13)	0.0008 (13)	0.0016 (12)

### Geometric parameters (Å, °)

**Table:**

F1—C4	1.349 (3)	C43—N44	1.340 (4)
C1—C6	1.395 (4)	N44—C45	1.357 (4)
C1—C2	1.396 (4)	C45—N47	1.360 (4)
C1—C7	1.484 (4)	C45—C46	1.408 (4)
C2—C3	1.382 (4)	N47—C48	1.458 (4)
C3—C4	1.381 (5)	C48—C49	1.510 (4)
C4—C5	1.376 (5)	C49—C50	1.384 (4)
C5—C6	1.378 (4)	C49—C54	1.392 (4)
C7—O8	1.214 (4)	C50—C51	1.386 (4)
C7—C9	1.539 (4)	C51—C52	1.383 (5)
C9—O10	1.215 (4)	C52—C53	1.374 (5)
C9—C11	1.491 (4)	C53—C54	1.391 (4)
C11—C12	1.392 (4)	N17—H17	0.9400
C11—C16	1.395 (4)	N47—H47	0.9400
C12—C13	1.391 (4)	C2—H2	0.9500
C13—N14	1.330 (4)	C3—H3	0.9500
N14—C15	1.355 (4)	C5—H5	0.9500
C15—N17	1.365 (4)	C6—H6	0.9500
C15—C16	1.400 (4)	C12—H12	0.9500
N17—C18	1.458 (4)	C13—H13	0.9500
C18—C19	1.513 (4)	C16—H16	0.9500
C19—C24	1.387 (4)	C18—H18B	0.9900
C19—C20	1.396 (4)	C18—H18A	0.9900
C20—C21	1.387 (4)	C20—H20	0.9500
C21—C22	1.393 (5)	C21—H21	0.9500
C22—C23	1.369 (5)	C22—H22	0.9500
C23—C24	1.385 (4)	C23—H23	0.9500
F2—C34	1.354 (3)	C24—H24	0.9500
C31—C36	1.391 (4)	C33—H33	0.9500
C31—C32	1.404 (4)	C35—H35	0.9500
C31—C37	1.474 (4)	C36—H36	0.9500
C32—C33	1.385 (5)	C42—H42	0.9500
C33—C34	1.369 (5)	C43—H43	0.9500
C34—C35	1.372 (4)	C46—H46	0.9500
C35—C36	1.385 (4)	C48—H48A	0.9900
C37—O38	1.213 (4)	C48—H48B	0.9900
C37—C39	1.535 (4)	C50—H50	0.9500
C39—O40	1.215 (4)	C51—H51	0.9500
C39—C41	1.495 (4)	C52—H52	0.9500
C41—C46	1.379 (4)	C53—H53	0.9500
C41—C42	1.393 (4)	C54—H54	0.9500
C42—C43	1.375 (4)		
			
C6—C1—C2	119.4 (3)	C49—C50—C51	120.5 (3)
C6—C1—C7	122.8 (2)	C52—C51—C50	120.3 (3)
C2—C1—C7	117.8 (3)	C53—C52—C51	119.9 (3)
C3—C2—C1	120.8 (3)	C52—C53—C54	120.0 (3)
C4—C3—C2	117.9 (3)	C53—C54—C49	120.6 (3)
F1—C4—C5	119.1 (3)	C15—N17—H17	116.00
F1—C4—C3	118.2 (3)	C18—N17—H17	115.00
C5—C4—C3	122.7 (3)	C45—N47—H47	115.00
C4—C5—C6	119.0 (3)	C48—N47—H47	121.00
C5—C6—C1	120.1 (3)	C3—C2—H2	120.00
O8—C7—C1	122.7 (3)	C1—C2—H2	120.00
O8—C7—C9	118.5 (3)	C4—C3—H3	121.00
C1—C7—C9	118.5 (2)	C2—C3—H3	121.00
O10—C9—C11	122.8 (3)	C4—C5—H5	120.00
O10—C9—C7	117.0 (3)	C6—C5—H5	121.00
C11—C9—C7	120.0 (3)	C1—C6—H6	120.00
C12—C11—C16	119.6 (3)	C5—C6—H6	120.00
C12—C11—C9	123.7 (3)	C13—C12—H12	122.00
C16—C11—C9	116.7 (3)	C11—C12—H12	122.00
C13—C12—C11	116.9 (3)	N14—C13—H13	117.00
N14—C13—C12	125.0 (3)	C12—C13—H13	118.00
C13—N14—C15	117.9 (2)	C11—C16—H16	121.00
N14—C15—N17	115.9 (3)	C15—C16—H16	120.00
N14—C15—C16	121.6 (3)	H18A—C18—H18B	108.00
N17—C15—C16	122.5 (3)	C19—C18—H18A	109.00
C11—C16—C15	119.0 (3)	C19—C18—H18B	109.00
C15—N17—C18	120.8 (2)	N17—C18—H18A	109.00
N17—C18—C19	110.7 (2)	N17—C18—H18B	110.00
C24—C19—C20	118.7 (3)	C21—C20—H20	120.00
C24—C19—C18	120.3 (3)	C19—C20—H20	119.00
C20—C19—C18	121.1 (3)	C20—C21—H21	120.00
C21—C20—C19	120.8 (3)	C22—C21—H21	120.00
C20—C21—C22	119.5 (3)	C23—C22—H22	120.00
C23—C22—C21	119.9 (3)	C21—C22—H22	120.00
C22—C23—C24	120.8 (3)	C22—C23—H23	120.00
C23—C24—C19	120.3 (3)	C24—C23—H23	120.00
C36—C31—C32	119.4 (3)	C19—C24—H24	120.00
C36—C31—C37	121.6 (3)	C23—C24—H24	120.00
C32—C31—C37	119.0 (3)	C31—C32—H32	120.00
C33—C32—C31	120.2 (3)	C33—C32—H32	120.00
C34—C33—C32	118.1 (3)	C32—C33—H33	121.00
F2—C34—C33	118.2 (3)	C34—C33—H33	121.00
F2—C34—C35	118.0 (3)	C34—C35—H35	121.00
C33—C34—C35	123.7 (3)	C36—C35—H35	121.00
C34—C35—C36	118.0 (3)	C31—C36—H36	120.00
C35—C36—C31	120.5 (3)	C35—C36—H36	120.00
O38—C37—C31	124.1 (3)	C41—C42—H42	121.00
O38—C37—C39	117.3 (3)	C43—C42—H42	121.00
C31—C37—C39	118.4 (2)	N44—C43—H43	118.00
O40—C39—C41	123.1 (3)	C42—C43—H43	118.00
O40—C39—C37	117.6 (3)	C41—C46—H46	121.00
C41—C39—C37	119.2 (2)	C45—C46—H46	121.00
C46—C41—C42	120.3 (3)	N47—C48—H48A	109.00
C46—C41—C39	117.9 (3)	N47—C48—H48B	109.00
C42—C41—C39	121.7 (3)	C49—C48—H48A	109.00
C43—C42—C41	117.3 (3)	C49—C48—H48B	109.00
N44—C43—C42	124.3 (3)	H48A—C48—H48B	108.00
C43—N44—C45	118.2 (2)	C49—C50—H50	120.00
N44—C45—N47	116.0 (2)	C51—C50—H50	120.00
N44—C45—C46	121.2 (3)	C50—C51—H51	120.00
N47—C45—C46	122.8 (3)	C52—C51—H51	120.00
C41—C46—C45	118.6 (3)	C51—C52—H52	120.00
C45—N47—C48	121.1 (2)	C53—C52—H52	120.00
N47—C48—C49	111.3 (2)	C52—C53—H53	120.00
C50—C49—C54	118.8 (3)	C54—C53—H53	120.00
C50—C49—C48	121.3 (3)	C49—C54—H54	120.00
C54—C49—C48	119.9 (3)	C53—C54—H54	120.00
			
C13—N14—C15—C16	−1.7 (4)	C18—C19—C24—C23	−178.4 (3)
C15—N14—C13—C12	−0.9 (4)	C24—C19—C20—C21	−2.6 (5)
C13—N14—C15—N17	177.3 (3)	C19—C20—C21—C22	0.9 (5)
C18—N17—C15—N14	172.6 (3)	C20—C21—C22—C23	0.6 (6)
C18—N17—C15—C16	−8.5 (4)	C21—C22—C23—C24	−0.4 (6)
C15—N17—C18—C19	174.1 (3)	C22—C23—C24—C19	−1.3 (5)
C45—N44—C43—C42	0.4 (4)	C32—C31—C36—C35	−1.1 (5)
C43—N44—C45—C46	1.2 (4)	C32—C31—C37—O38	−5.4 (5)
C43—N44—C45—N47	−177.5 (3)	C37—C31—C32—C33	−177.2 (3)
C45—N47—C48—C49	−173.5 (3)	C36—C31—C32—C33	2.5 (5)
C48—N47—C45—C46	7.2 (4)	C36—C31—C37—O38	175.0 (3)
C48—N47—C45—N44	−174.1 (3)	C36—C31—C37—C39	−10.1 (4)
C2—C1—C6—C5	−1.0 (4)	C32—C31—C37—C39	169.5 (3)
C2—C1—C7—C9	−161.5 (3)	C37—C31—C36—C35	178.5 (3)
C2—C1—C7—O8	12.6 (4)	C31—C32—C33—C34	−0.8 (5)
C6—C1—C7—C9	19.6 (4)	C32—C33—C34—C35	−2.3 (5)
C7—C1—C2—C3	−177.7 (3)	C32—C33—C34—F2	178.4 (3)
C6—C1—C2—C3	1.3 (5)	F2—C34—C35—C36	−177.1 (3)
C7—C1—C6—C5	177.8 (3)	C33—C34—C35—C36	3.6 (5)
C6—C1—C7—O8	−166.3 (3)	C34—C35—C36—C31	−1.8 (5)
C1—C2—C3—C4	−0.7 (5)	C31—C37—C39—C41	126.0 (3)
C2—C3—C4—C5	−0.1 (6)	C31—C37—C39—O40	−57.3 (4)
C2—C3—C4—F1	−178.7 (3)	O38—C37—C39—O40	118.0 (3)
C3—C4—C5—C6	0.3 (6)	O38—C37—C39—C41	−58.8 (4)
F1—C4—C5—C6	178.9 (3)	O40—C39—C41—C42	176.1 (3)
C4—C5—C6—C1	0.3 (5)	C37—C39—C41—C46	176.7 (3)
C1—C7—C9—O10	41.8 (4)	C37—C39—C41—C42	−7.4 (4)
C1—C7—C9—C11	−142.6 (3)	O40—C39—C41—C46	0.2 (4)
O8—C7—C9—C11	43.0 (4)	C39—C41—C42—C43	−174.7 (3)
O8—C7—C9—O10	−132.6 (3)	C46—C41—C42—C43	1.1 (4)
C7—C9—C11—C12	13.7 (5)	C42—C41—C46—C45	0.4 (4)
O10—C9—C11—C16	7.2 (5)	C39—C41—C46—C45	176.3 (3)
C7—C9—C11—C16	−168.2 (3)	C41—C42—C43—N44	−1.5 (5)
O10—C9—C11—C12	−171.0 (3)	N44—C45—C46—C41	−1.6 (4)
C12—C11—C16—C15	0.3 (5)	N47—C45—C46—C41	177.1 (3)
C9—C11—C16—C15	−178.0 (3)	N47—C48—C49—C50	51.5 (4)
C9—C11—C12—C13	175.5 (3)	N47—C48—C49—C54	−130.3 (3)
C16—C11—C12—C13	−2.6 (4)	C48—C49—C50—C51	179.4 (3)
C11—C12—C13—N14	3.1 (4)	C54—C49—C50—C51	1.2 (5)
N17—C15—C16—C11	−176.9 (3)	C48—C49—C54—C53	−179.3 (3)
N14—C15—C16—C11	2.0 (4)	C50—C49—C54—C53	−1.1 (5)
N17—C18—C19—C24	129.3 (3)	C49—C50—C51—C52	−0.7 (5)
N17—C18—C19—C20	−51.8 (4)	C50—C51—C52—C53	0.0 (5)
C20—C19—C24—C23	2.7 (5)	C51—C52—C53—C54	0.2 (5)
C18—C19—C20—C21	178.6 (3)	C52—C53—C54—C49	0.4 (5)

### Hydrogen-bond geometry (Å, °)

**Table:**

*D*—H···*A*	*D*—H	H···*A*	*D*···*A*	*D*—H···*A*
N17—H17···N44	0.94	2.10	3.037 (3)	174
N47—H47···N14	0.94	2.15	3.087 (4)	172
